# Habitual Drug Intake Is Hard to Change: On the Discussion About Habits and Compulsions in Drug Addiction

**DOI:** 10.1111/adb.70081

**Published:** 2025-09-26

**Authors:** Andreas Heinz, Stefan Gutwinski, Eva Friedel, Nadja Samia Bahr, Rainer Spanagel, Gaetano Di Chiara

**Affiliations:** ^1^ Department of Psychiatry and Psychotherapy Universität Tübingen Tübingen Germany; ^2^ German Center for Mental Health (DZPG) Tübingen Germany; ^3^ Department of Psychiatry and Psychotherapy TU Dresden Dresden Germany; ^4^ Department of Psychiatry and Neuroscience CCM, NeuroCure Clinical Research Center, Berlin Institute of Health CCM Charité – Universitätsmedizin Berlin, Freie Universität Berlin, Humboldt‐Universität zu Berlin Berlin Germany; ^5^ Department of Psychosomatic Medicine Charité – Universitätsmedizin Berlin Berlin Germany; ^6^ German Center for Mental Health (DZPG) Berlin‐Potsdam Germany; ^7^ Institute of Psychopharmacology, Central Institute of Mental Health, Medical Faculty Mannheim Heidelberg University Mannheim Germany; ^8^ German Center for Mental Health (DZPG) Mannheim Germany; ^9^ Department of Biomedical Sciences University of Cagliari, Cittadella Universitaria di Monserrato, Monserrato Cagliari Italy; ^10^ Neuroscience Institute National Research Council of Italy (CNR) Cagliari Italy

In our recent opinion paper in *Addiction Biology* ‘Does compulsion explain addiction’? [1], we criticize that compulsive behaviour as defined primarily from the perspective of animal experimentation falls short of the clinical phenomenon. In particular, we argue that animal models of compulsion with immediate punishment do not adequately reflect long‐term negative outcomes in human addiction. We also discuss that compulsive behaviour in OCD and addiction differ phenomenologically. Finally, we opine that human drug use can become a habit, but this does not imply a general loss of goal‐directed decision‐making. Instead, we claim that ‘individuals with drug addiction are neither automatons carrying out habitual behaviour without cognitive control nor merely individuals making bad choices’. Altogether, we conclude that habit formation can contribute to drug intake, as long as habitual behaviour is regarded as dimensionally but not categorically different from goal‐directed decision‐making and modifiable by human cognition. This statement is supported by studies from the ReCoDe consortium [2] and by a recent meta‐analysis of the habit construct in animal models of alcohol use disorder [3], which did not indicate a strict habit‐goal dichotomy. Instead, this meta‐analysis suggests (i) a nuanced transition between goal directed and habitual decision making and (ii) distinguishes habitual responding from compulsivity [3].

Karen Ersche [4] criticizes our paper and suggests that OCD and addiction have more in common than we describe. She states that positive and negative reinforcement are associated with both drug intake and OCD, with which we agree. However, we disagree with Ersche's definition of compulsion as ‘ongoing actions that have become inappropriate to the immediate context’, because this definition is too broad and would, for example, include behaviours observed in bipolar and psychotic disorders unrelated to both OCD and addiction.

At one point, Ersche seems to confuse our criticism of the concept of compulsion in addiction with Lee Hogarth's criticism of the habit construct when she directly refers to one of his publications [5] which, however, was not cited in our opinion paper. In fact, we are not simply ‘rejecting’ the habit theory of addiction. Instead, we argue that the dual‐system approach posits a categorical distinction between a habit and a goal‐directed brain system, whereas nuanced transitions between these behavioural control systems occur in animals [3] and humans [6]. Thus, we argue that there is no overall loss of goal‐directed behaviour, nor a general over‐reliance on habits in individuals with drug addiction, but drug use itself can become a ‘habit’, that is, a bias toward drug‐seeking in certain (e.g., stressful) circumstances [7].

Lee Hogarth [8] challenges our claim that habit formation contributes to drug addiction. He understands habits within the narrow confines of the habit/compulsion theory of addiction, where habits are defined as ‘representations of stimulus–response links that do not refer to goals’ [9]. Hogarth claims that this habit theory constructs ‘drug users as intellectually impaired’ and thus ‘recapitulate[s] the social injustice of the racial intelligence era by prematurely attributing lower task performance to drug user group membership’. His argument rests on three points: (i) that it is racist to attribute lower cognitive test performance in racialized groups to ‘race’ while ignoring social disadvantage, (ii) that habit formation is a form of intellectual impairment and (iii) that, by analogy, attributing increased habit formation as intellectual impairment to individuals with drug use disorders while ignoring social disadvantage amounts to scientific racism. With respect to (i), Hogarth cites the findings of Weiss [10] and claims that social disparity fully explains the difference in IQ test results between racialized groups. However, this is not true. Weiss [10] suggests that gaps remaining in cognitive performance after correcting for social disparities are due to ‘historical discrimination’, which includes racism. Therefore, argument (i) of Hogarth needs to be revised: It is indeed racist to attribute lower cognitive test performance in racialized groups to ‘race’ while ignoring social disadvantage AND the impact of racism itself on cognitive test performance. In fact, racism and other forms of historically embedded discrimination can induce severe stress and have profound negative effects on mental health [11, 12] and cognitive performance [13]. Such effects are then reified by the ‘race’ term, which falsely postulates categorical rather than gradual differences among populations [14]. Regarding argument (ii), we agree with Hogarth that some theories of addictive behaviour stigmatize patients and wrongly attribute cognitive effects of social exclusion and discrimination to genetic causes [15]. However, we do not view habitual behaviour as an intellectual impairment.

Habits are useful for all human beings: We could not drive a car and have a complex discussion at the same time without habit formation. In accordance with a recent meta‐analysis [3], we observed gradual differences between habitual and goal‐directed decision making and did not find a general deficit in goal‐directed decision‐making in persons with alcohol use disorders (AUD) [16]. Emphasizing that individuals with drug addiction are not ‘automatons carrying out habitual behaviour without cognitive control’ [1] does not rule out that specific behaviour patterns (including e.g., individually disadvantageous stress coping mechanisms) can become habitual, as evinced by the alcohol approach bias [17]. Individuals with AUD can change this approach bias with intensive training, indicating that individuals with habitual drug use patterns may need specific support to ‘break the habit’ Hogarth's claim that attributing habitual behaviour to individuals with substance use disorders amounts to labeling them as intellectually impaired may be motivated by theories that explain habit formation as the result of dysfunction of a higher cognitive control centre or goal‐directed system. However, in our opinion article [1], we explicitly rejected reifying behaviour patterns such as goal‐directed behaviour into ‘systems’, and we understand habit formation as an important ability. Therefore, argument (ii) of Hogarth is not in line with our point of view. With respect to argument (iii), we agree with Hogarth that social disadvantage, exclusion and discrimination play an important role in the predisposition to addiction [18, 19]. Moreover, we do not claim that habit formation is a pathological process that can only occur when goal‐directed behaviour is impaired due to neurotoxic effects of alcohol. This being said, alcohol intake can cause neurotoxicity, which in turn can contribute to cognitive impairments that require support and therapy [20, 21, 22, 23, 24]. Ignoring the effects of drugs and drug‐related mental disorders on motivation and cognition risks blaming the victims for their problems [15]. This unintended consequence is illustrated by Thomas Szasz [25], who reasoned that considering his postulated absence of valid concepts of mental illness, individuals do not have mental disorders but are instead just lazy and should therefore lose social support. In summary, we advise against trivializing the important topic of racism by inadequately equating studies measuring the neurotoxic and neurobiological effects of drugs of abuse on cognition with racist theories of group inferiority. Instead, the effects of racism on cognition and health should be taken seriously in their own right [26, 27, 28].

We agree with Ersche [4] and Hogarth [8] that in order to avoid stigmatization and discrimination, we need to reflect on our disease categories and their implications.

Our disease concepts must avoid stigmatizing individuals who use drugs, without denying the negative medical consequences of drug use and without excluding individuals with drug addiction from receiving the necessary support within the medical care system. We concur with Ersche's assertion that while a critical reflection of theories of addictive behaviour is good scientific practice, it necessitates the development of a more comprehensive theory to take its place. What the field of addiction critically requires is a unifying theory that integrates consistent findings across all levels of analysis—from genetic and molecular to neuronal, circuit, behavioural, and social aspects of addiction. This is an ambitious endeavour that can only be achieved through an open‐minded approach by numerous experts.

## Conflicts of Interest

The authors declare no conflicts of interest.

## References

[1] A. Heinz , S. Gutwinski , N. S. Bahr , R. Spanagel , and G. Di Chiara , “Does Compulsion Explain Addiction?,” Addiction Biology 29, no. 4 (2024): e13379, 10.1111/adb.13379. PMC11001268 38588458

[2] R. Spanagel , P. Bach , T. Banaschewski , et al., “The ReCoDe Addiction Research Consortium: Losing and Regaining Control Over Drug Intake‐Findings and Future Perspectives,” Addiction Biology 29, no. 7 (2024): e13419, 10.1111/adb.13419. PMC11215792 38949209

[3] F. Giannone , C. Ebrahimi , T. Endrass , A. C. Hansson , F. Schlagenhauf , and W. H. Sommer , “Bad Habits‐Good Goals? Meta‐Analysis and Translation of the Habit Construct to Alcoholism,” Translational Psychiatry 14, no. 1 (2024): 298, 10.1038/s41398-024-02965-1. 39030169 PMC11271507

[4] K. D. Ersche , “Defending and Defining Compulsive Behaviour in Addiction,” Addiction Biology 29, no. 8 (2024): e13427, 10.1111/adb.13427. PMC11317886 39132705

[5] L. Hogarth , “Addiction is Driven by Excessive Goal‐Directed Drug Choice Under Negative Affect: Translational Critique of Habit and Compulsion Theory,” Neuropsychopharmacology 45, no. 5 (2020): 720–735, 10.1038/s41386-020-0600-8. 31905368 PMC7265389

[6] L. Deserno , Q. J. M. Huys , R. Boehme , et al., “Ventral Striatal Dopamine Reflects Behavioral and Neural Signatures of Model‐Based Control During Sequential Decision Making,” National Academy of Sciences of the United States of America 112, no. 5 (2015): 1595–1600, 10.1073/pnas.1417219112. PMC4321318 25605941

[7] K. Chen , B. Hollunder , M. Garbusow , M. Sebold , and A. Heinz , “The Physiological Responses to Acute Stress in Alcohol‐Dependent Patients: A Systematic Review,” European Neuropsychopharmacology 41 (2020): 115, 10.1016/j.euroneuro.2020.09.003. 32994116

[8] L. Hogarth , “Motivated Reasoning and Scientific Racism in Compulsion Theory of Human Addiction: Methodological Framework to Promote Social Justice,” Addiction Biology 29, no. 8 (2024): e13435, 10.1111/adb.13435. 39188063 PMC11347614

[9] T. W. Robbins and R. M. Costa , “Habits,” Current Biology 27, no. 22 (2017): R1200–R1206, 10.1016/j.cub.2017.09.060. 29161553

[10] L. G. Weiss and D. H. Saklofske , “Mediators of IQ Test Score Differences Across Racial and Ethnic Groups: The Case for Environmental and Social Justice,” Personality and Individual Differences 161 (2020): 109962, 10.1016/j.paid.2020.109962.

[11] F. B. Lazaridou , A. Heinz , D. Schulze , and D. Bhugra , “Racialised Identity, Racism and the Mental Health of Children and Adolescents,” International Review of Psychiatry 35, no. 3–4 (2023): 277–288, 10.1080/09540261.2023.2181059. 37267023

[12] C. Emmer , J. Dor , and J. Mata , “The Immediate Effect of Discrimination on Mental Health: A Meta‐Analytic Review of the Causal Evidence,” Psychological Bulletin 150, no. 3 (2024): 215–252, 10.1037/bul0000419. 38330346

[13] J. A. Kaminski , F. Schlagenhauf , M. Rapp , et al., “Epigenetic Variance in Dopamine D2 Receptor: A Marker of IQ Malleability?,” Translational Psychiatry 8, no. 1 (2018), 10.1038/s41398-018-0222-7. PMC6117339 30166545

[14] A. Heinz , D. J. Müller , S. Krach , M. Cabanis , and U. P. Kluge , “The Uncanny Return of the Race Concept,” Frontiers in Human Neuroscience 8 (2014): 8, 10.3389/fnhum.2014.00836. PMC4219449 25408642

[15] J. Rothenberg and A. Heinz , “Meddling With Monkey Metaphors — Capitalism and the Threat of Impulsive Desires,” Social Justice 25, no. 2 (72) (1998): 44–64, http://www.jstor.org/stable/29767070.

[16] M. Sebold , S. Nebe , M. Garbusow , et al., “When Habits Are Dangerous: Alcohol Expectancies and Habitual Decision Making Predict Relapse in Alcohol Dependence,” Biological Psychiatry 82, no. 11 (2017): 847–856, 10.1016/j.biopsych.2017.04.019. 28673442

[17] R. W. Wiers , C. Eberl , M. Rinck , E. S. Becker , and J. Lindenmeyer , “Retraining Automatic Action Tendencies Changes Alcoholic Patients' Approach Bias for Alcohol and Improves Treatment Outcome,” Psychological Science 22, no. 4 (2011): 490–497, 10.1177/0956797611400615. 21389338

[18] L. Brandt , S. Liu , C. Heim , and A. Heinz , “The Effects of Social Isolation Stress and Discrimination on Mental Health,” Translational Psychiatry 12, no. 1 (2022), 10.1038/s41398-022-02178-4. PMC9490697 36130935

[19] D. Marbin , S. Gutwinski , S. Schreiter , and A. Heinz , “Perspectives in Poverty and Mental Health,” Frontiers in Public Health 10 (2022): 975482, 10.3389/fpubh.2022.975482. PMC9386343 35991010

[20] K. Stavro , J. Pelletier , and S. Potvin , “Widespread and Sustained Cognitive Deficits in Alcoholism: A Meta‐Analysis,” Addiction Biology 18, no. 2 (2012): 203–213, 10.1111/j.1369-1600.2011.00418.x. 22264351

[21] N. M. Zahr and A. Pfefferbaum , “Alcohol's Effects on the Brain: Neuroimaging Results in Humans and Animal Models,” Alcohol Research: Current Reviews 38, no. 2 (2017): 183–206. 28988573 10.35946/arcr.v38.2.04 PMC5513685

[22] C. Harper , “The Neurotoxicity of Alcohol,” Human & Experimental Toxicology 26, no. 3 (2007): 251–257, 10.1177/0960327107070499. 17439928

[23] S. De Santis , P. Bach , L. Pérez‐Cervera , et al., “Microstructural White Matter Alterations in Men With Alcohol Use Disorder and Rats With Excessive Alcohol Consumption During Early Abstinence,” JAMA Psychiatry 76, no. 7 (2019): 749–758, 10.1001/jamapsychiatry.2019.0318. 30942831 PMC6583663

[24] W. H. Sommer and S. Canals , “Alcohol‐Induced Changes in Brain Microstructure: Uncovering Novel Pathophysiological Mechanisms of AUD Using Translational DTI in Humans and Rodents,” Current Topics in Behavioral Neurosciences (2025), 10.1007/7854_2025_585. 40360929

[25] T. Szasz , Cruel Compassion: Psychiatric Control of Society's Unwanted (Syracuse University Press, 1998).

[26] J. I. Fulton , H. Singh , O. Pakkal , E. M. Uleryk , and L. M. Nelson , “Community‐Based Culturally Tailored Education Programmes for Black Adults With Cardiovascular Disease, Diabetes, Hypertension and Stroke: A Systematic Review Protocol of Primary Empirical Studies,” BMJ Open 12, no. 6 (2022): e059883, 10.1136/bmjopen-2021-059883. PMC9189819 35688600

[27] F. Lazaridou and S. Fernando , “Deconstructing Institutional Racism and the Social Construction of Whiteness: A Strategy for Professional Competence Training in Culture and Migration Mental Health,” Transcultural Psychiatry 59, no. 2 (2022): 175–187, 10.1177/13634615221087101. 35373653 PMC9026223

[28] L. Hertner , P. Stylianopoulos , A. Heinz , U. Kluge , I. Schäfer , and S. Penka , “Substance (Mis)Use Among Refugees as a Matter of Social Ecology: Insights Into a Multi‐Site Rapid Assessment in Germany,” Conflict and Health 17, no. 1 (2023), 10.1186/s13031-023-00499-9. PMC9850330 36658646

